# CBCT data relevant in treatment planning for immediate mandibular molar implant placement

**DOI:** 10.34172/japid.025.3722

**Published:** 2025-04-14

**Authors:** Maziar Ebrahimi Dastgurdi, Douglas Deporter, Max Xia, Mohammad Ketabi

**Affiliations:** ^1^Discipline of Endodontics, Faculty of Dentistry, University of Toronto, Toronto, ON, Canada; ^2^Discipline of Periodontology, Faculty of Dentistry, University of Toronto, Toronto, ON, Canada; ^3^Department of Periodontics, Faculty of Dentistry, IAU, Isfahan Branch (Khorasgan), Isfahan, Iran

**Keywords:** CBCT, Immediate implant, Mandible, Molar

## Abstract

**Background.:**

Immediate molar implants (IMIs) have been shown to provide an effective treatment, but their placement comes with potential anatomically related risks.

**Methods.:**

CBCTs of>400 dental sites were analyzed for key anatomical features at mandibular molar sites that can impact the placement of IMIs. Features measured included distances from each molar furcation to points risking lingual plate perforation or inferior alveolar nerve (IAC) damage, distances from molar root apices to IAC, mesiodistal and buccolingual widths of molar inter-septal bone (ISB), and thicknesses of buccal and lingual cortical plates at first and second mandibular molar sites.

**Results.:**

Distances from molar furcations to contact with lingual cortical plates and to IAC decreased significantly from mesial to distal, as did distances from root apices to the mandibular canal. Both buccolingual and mesiodistal ISB widths and thicknesses of buccal and lingual cortical plates increased mesiodistally. Buccolingual ISB widths were largest coronally for both molar sites and decreased apically. The reverse was found with mesiodistal septal ISB widths, which increased coronoapically.

**Conclusion.:**

Risks of lingual perforations or IAC damage were significantly greater at second molars vs. first molars. The ability to place IMIs in ISB at first molars was estimated to be>twice as often as at second molars. Maximal implant lengths for IMIs placed in the furcal bone should not exceed 10 mm.

## Introduction

 The immediate replacement of failed mandibular molar teeth with dental implants has become increasingly common in recent years to streamline implant protocols, satisfy patient demands, and reduce the commonly dramatic shrinkage in alveolar ridge dimensions after extraction with delayed implant placement.^1^ However, “immediacy” in molar replacement does require careful pre-treatment planning to avoid complications and failure by ensuring that immediate molar implants (IMIs) are placed with ideal positioning in three dimensions (buccolingual, mesiodistal, and apicocoronal).^2^ This is most commonly achieved by initiating osteotomies into the molar ISB either before tooth removal^3,4^ or afterward, with innovative protocols like the recently proposed technique using specialized burs run counterclockwise to expand the bone volume available.^5^

 Multiple anatomical issues need consideration before opting for an IMI at mandibular molar sites. Serious risk factors include bur perforation of the lingual cortical plate and/or damage to the inferior alveolar nerve (IAN). The posterior mandible often presents with a significant lingual concavity at either first or second molar sites with higher prevalence generally at the latter^6,7^ particularly in southeast Asians.^8^ Regarding risks of damaging the IAN, it has long been held that at least 2 mm of native bone should be left undisturbed above the canal if osteotomy drilling is to avoid nerve damage.^9^ Knowing both buccolingual and mesiodistal dimensions of available ISB is also valuable in deciding whether it is sufficient to stabilize an IMI.^10^ In addition, if the ISB is not a suitable site and consideration is being given to using one or other of the molar root sockets for an IMI, it will be important to know the distances from root apices to IAN and the condition and thicknesses of the buccal and lingual cortices of both root sockets.

## Methods

 Cone-beam computed tomographies (CBCTs) collected from 412 dental sites in 204 dentulous patients were available for analysis in this study. All patients signed a consent form permitting their CBCT images to be used for this project.

 All images were measured twice within a 4-week interval to assess examiner reliability. Examiner reliability was evaluated at a statistical significance of *P* < 0.05.

 To be included, patients needed to be > 18 years of age (21‒74 years) and to have at least two occluding mandibular posterior teeth (premolar and/or molar), at least one of which was a fully erupted molar with fully formed root apices. Exclusion criteria included radiographic evidence of periodontal bone loss, infection, severe root resorption or periapical pathology, history of previous surgical interventions at the selected teeth, presence of metal restorations affecting CBCT quality, and/or a history of the use of medications that could have affected the skeletal system.

**Figure 1:**
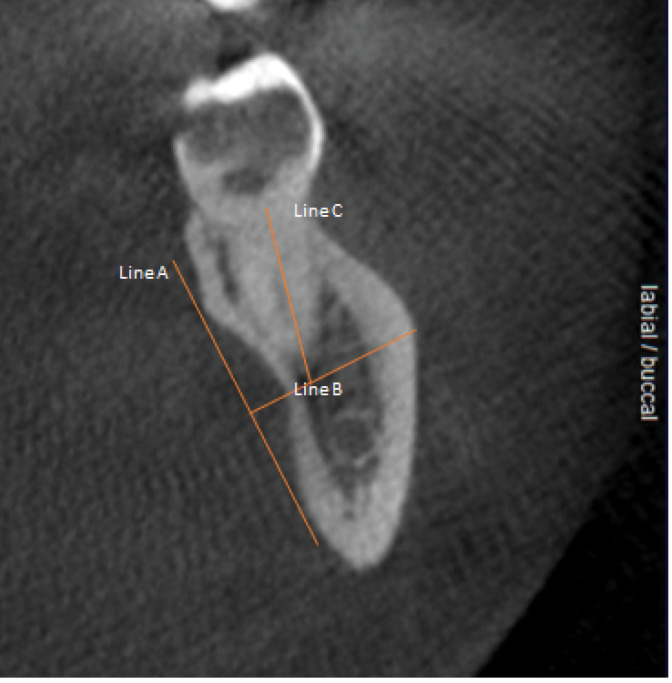
Sagittal sections of relevant CBCTs were marked as shown. Line “A” was used to define the existing concavity, while Line “B” was traced perpendicular to “A”. Line “C” was traced through the middle of a CT slice corresponding to the location of the tooth’s furcation and aligning with the long axis of the tooth and represented the distance from furcation to the level of the deepest point of the lingual cavity, i.e., the point where a perforation of the lingual cortical plate was a risk

**Figure 2:**
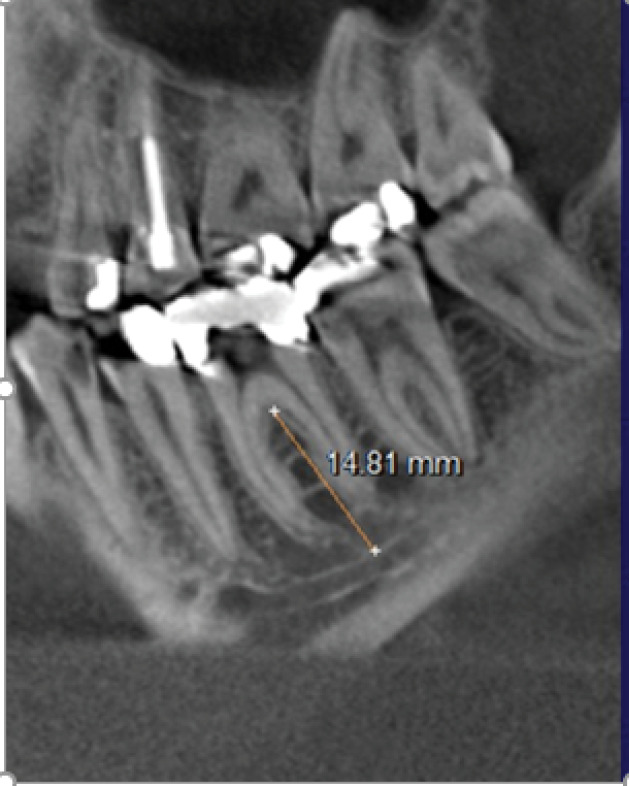
The distance from furcation to the inferior alveolar canal (IAC) was measured in the middle-most coronal CBCT slice

**Figure 3:**
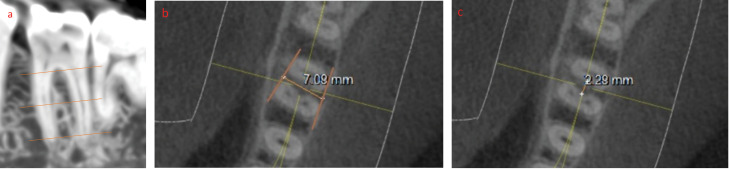
Measurements of the buccolingual and mesiodistal dimensions of ISB were taken at three levels. (a) A crestal measurement was recorded at 0.5 mm apical to the molar furcation. (b) An apical measurement was recorded at 0.5 mm coronal to the line connecting the apices of the two shortest roots. (c) A middle measurement was recorded midway between the crestal and apical levels

**Figure 4:**
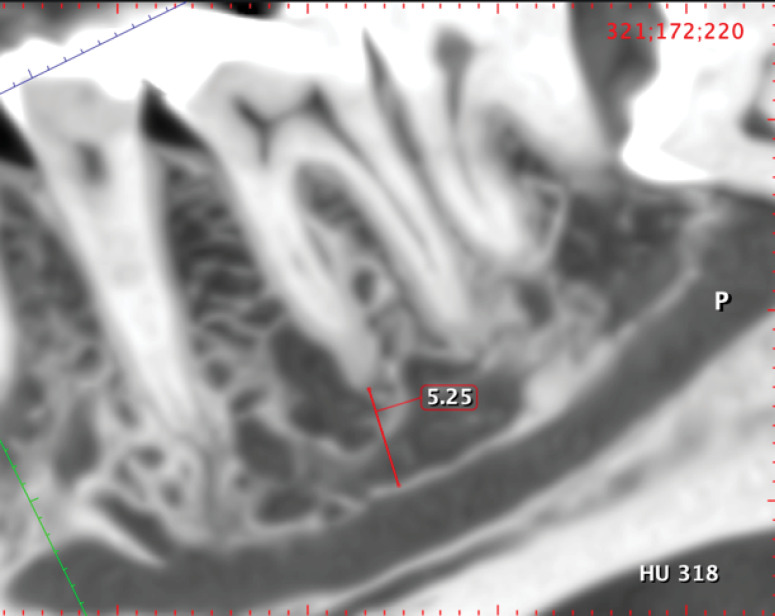
The apical distances from mesial and distal molar roots to the IAN were recorded

**Figure 5:**
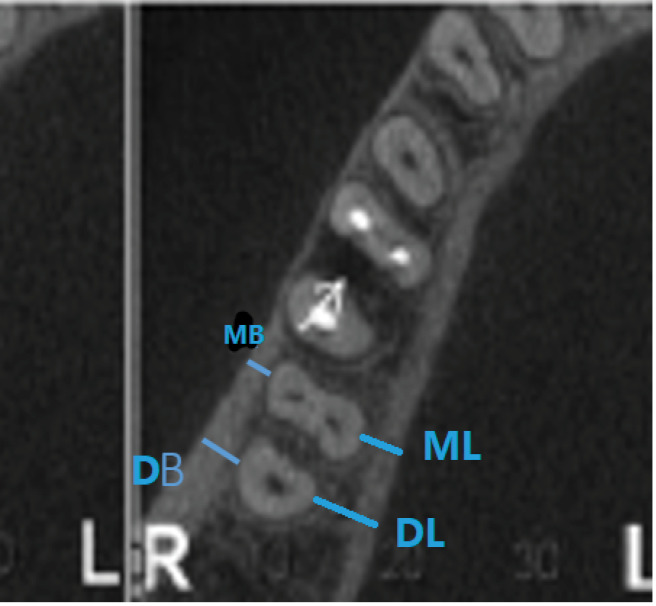
The thicknesses of buccal and lingual cortical plates were measured like those of the ISB at three levels (crestal, mid-root, and apical)

 Measurements made at mandibular first and second molars included the following:

- *The distance from each molar furcation to the deepest point of any associated lingual concavity:* To determine the distance from molar furcation to the deepest point of any relevant mandibular lingual concavity, appropriate sagittal CBCT sections were marked with three horizontal lines (Figure 1). Line “A” was used to define and contain the extent of each concavity, while line “B” was made perpendicular to line “A” through the deepest point of each concavity. Finally, line “C” ran through the middle and followed the coronoapical direction of the related tooth root and represented the distance from furcation to the level of the deepest point of the lingual cavity, i.e., the point where a perforation of the lingual cortical plate was a risk.
- *The distance from each molar furcation to the mandibular canal (IAN)* (Figure 2).
- *The mesiodistal and buccolingual dimensions of the molar inter-septal bone (ISB):* Measurements were taken of the buccolingual and mesiodistal dimensions of ISB at three levels (Figure 3): (a) a crestal measurement 0.5 mm apical to the molar furcation; (b) an apical measurement 0.5 mm coronal to the line connecting the apices of the two roots; and (c) a middle measurement midway between the crestal and apical levels.
- *The apical distance from mesial and distal molar roots to the IAN* (Figure 4).
- *The thicknesses of the buccal and the lingual cortical plates adjacent to both the mesial and distal roots of each molar tooth* (Figure 5).

###  Statistical analyses 

 Statistical analyses were conducted to explore various aspects of our dataset. The homogeneity of variances across groups was initially evaluated using Levene’s test. Depending on the results, Student’s t-test or Welch’s t-test was applied as deemed appropriate for comparing continuous variables within the first and second molars.

 Collinearity among the cortical thickness measurements for each type of molar was assessed using Pearson’s correlation analysis. Linear mixed models (LMMs) were used to handle the inherent correlation in the data. Random effects for subjects and fixed effects for different measurement locations were included in these models, allowing significant differences in the mean measurements across various locations to be detected.

 The chi-squared test was also used to compare specific variables between the first and second molars. Examiner reliability was assessed using the intraclass correlation coefficient (ICC), and consistency in the measurements was ensured. Statistical significance was established at a threshold of *P* < 0.05. All analyses, including the LMMs, were performed using SPSS 26.0 for Windows.

## Results

###  Distances from Molar furcation to the deepest point of any existing lingual concavity

**Figure 6:**
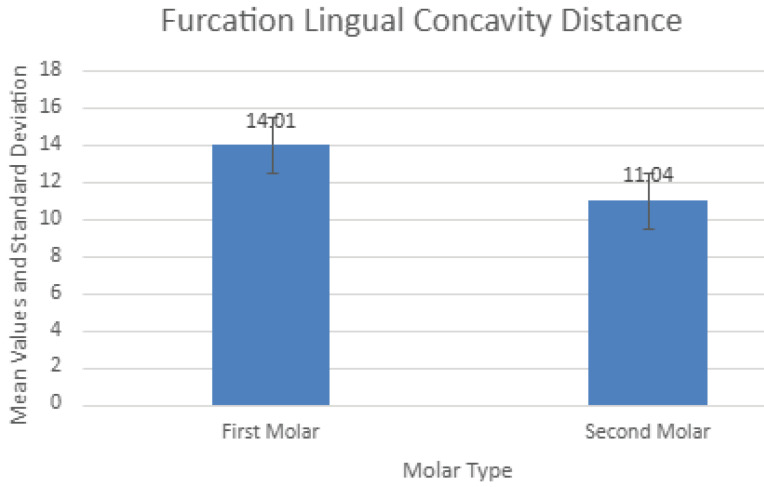
The mean distances from molar furcation to the deepest points of any lingual concavity

Figure 6 shows these distances for both mandibular first and second molars. The mean measurements were significantly (*P* < 0.001) different between the two molar locations (a mean of 14.01 mm for the first vs. 11.04 mm for the second), with a greater risk of perforating the lingual cortical plate at the second molar site.

###  Distances from molar furcation to the inferior alveolar canal (IAC)

**Figure 7:**
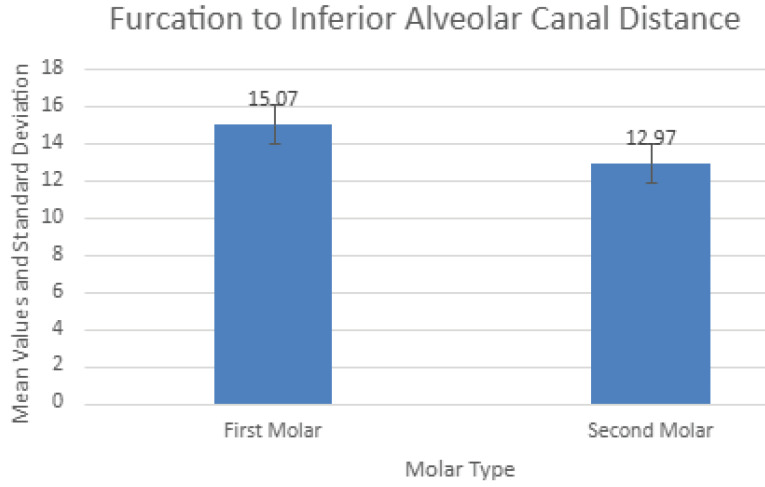
The mean distances from molar furcation to inferior alveolar canal

Figure 7 presents these measurements. Similar to the distances from furcation to the deepest level of associated lingual concavities, there were significantly (*P* < 0.001) different values for the two molars, with the second molars again showing smaller distances to the nerve canal.

###  Dimensions of inter-septal bone (ISB) 

Table 1 presents the mesiodistal and buccolingual dimensions of ISB for the two molar locations. All measurements were taken at three heights in the vertical plane, those being coronal (0.5 mm apical to the furcation), apical (at 0.5 mm coronal to a line connecting the apices of the two roots), and middle level taken at the mid-point between the other two measurements.

 As recorded, there were significant differences (*P* < 0.05) at all three measurement points between the first and second molars (both buccolingual and mesiodistal ISB widths).

 Widths were largest at the coronal-most level for both molar sites and decreased apically. The reverse was the situation with mesiodistal septal bone widths, which increased coronoapically.

###  Apical distances from mesial and distal molar root apices to the IAC

Table 2 shows these measurements. Distances were largest at the mesial root socket (5.52 mm, SD = 1.28 mm) of the first molar, decreasing posteriorly to the smallest value at the distal root of the second molar (3.84 mm, SD = 1.53 mm), and the differences were significant (*P* < 0.001 for the mesial roots of first vs. second molars and their distal roots as well (*P* < 0.0001).

###  Thicknesses of the buccal and lingual cortical plates adjacent to both the mesial and distal roots of each molar tooth

Table 3 presents the buccal and lingual cortical bone thickness measurements. Lingual thicknesses were always thicker than buccal ones. Thicknesses generally increased progressively from the mesial root socket of the first molar to the distal root socket of the second molar and coronoapically. There were significant differences (*P* < 0.001) between the mesial and distal root sockets of the mandibular first molars and between the two roots of the second molars (*P* < 0.01). Other comparisons showed significant differences between the distal roots of the first versus the mesial root sockets of the second molars (*P* < 0.001). For example, at mid-root measurement levels, the values were 2.3 ± 1.2 mm at the distobuccal aspect of the first molar vs. 4.8 ± 1.7 mm at the mesiobuccal aspect of the second molar sites (*P* < 0.001).

**Table 1:** Measurements of buccolingual and mesiodistal dimensions of inter-septal bone for both first and second mandibular molars recorded at three levels

**Measurement typ**e	**Abbreviation**	**First molar**	**Second molar**
		**Mean (SD)**	**Mean (SD)**
Coronal buccolingual distance	CBD	7.85 (2.85)	6.53 (3.00)
Middle buccolingual distance	MBD	6.75 (2.10)	5.91 (2.01)
Apical buccolingual distance	APD	5.22 (1.41)	4.57 (1.47)
Coronal mesiodistal distance	CMD	2.22 (0.76)	2.00 (0.41)
Middle mesiodistal distance	MMD	3.16 (0.95)	2.91 (1.31)
Apical mesiodistal distance	AMD	3.15 (0.68)	2.63 (0.48)

**Table 2:** Measurements of the distances from mesial and distal molar root apices to the IAN

**Measurement type**	**Abbreviation**	**First molar**	**Second molar**
		**Mean (SD)**	**Mean (SD)**
Mesial root apex to the inferior alveolar canal	MRAIAC	5.52 (1.28)	4.34 (1.74)
Distal root apex to the inferior alveolar canal	DRAIAC	5.24 (1.13)	3.84 (0.53)

**Table 3:** Measurements of the thicknesses of buccal and lingual cortices of the mesial and distal root sockets of the first and second mandibular molars taken at three levels (crestal, middle root, and apical)

**Teeth**	**Measurement level (Mean ± SD)**											
	**Coronal (1 mm apical to the CEJ)**				**Mid-root**				**Apical (1 mm coronal to the apex)**			
	**MB**	**DB**	**ML**	**DL**	**MB**	**DB**	**ML**	**DL**	**MB**	**DB**	**ML**	**DL**
First Molar	0.31 ± 0.1	0.43 ± 0.12	0.85 ± 0.32	0.92 ± 0.61	1.81 ± 0.94	2.33 ± 1.22	3.9 ± 0.96	3.76 ± 0.81	1.52 ± 0.78	2.08 ± 1.2	5.39 ± 0.88	5.8 ± 1.19
Second molar	1.03 ± 0.97	2.04 ± 0.92	1.1 ± 0.58	1.93 ± 0.47	4.78 ± 1.7	5.08 ± 1.64	2.35 ± 1.04	2.58 ± 1.31	5.1 ± 1.65	6.22 ± 1.68	4.41 ± 1.13	4.94 ± 1.27

## Discussion

**Figure 8:**
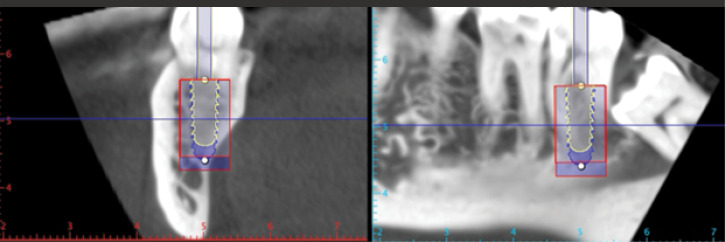
Placing a 10-mm-long immediate implant in the second mandibular molar position may risk lingual perforation and violation of the 2-mm safety zone to the mandibular canal. A shorter (8 mm) but wider implant may be indicated to avoid these complications

 CBCT is a valuable imaging technique in oral and maxillofacial surgery as it allows accurate diagnosis and treatment with dental implants using three-dimensional images without the financial burden and radiation exposure of conventional computed tomography (CT) scans. Its limitation is that it does not provide a detailed depiction of soft tissue conditions, which did not affect the current analyses of bony anatomy in the posterior mandible. Of all the risks of using IMIs to restore mandibular first and second molars, penetration of the lingual cortical plate and damage to the IAN are prime concerns (Figure 8). Mandibular second molars are more likely to have lingual undercuts (type “U” jaw anatomy) than first molars.^7,8^ Lingual perforation is estimated to have an occurrence of 1%‒2% only but can result in life-threatening sublingual hematomas,^11^ nerve damage, inflammation, and infection.^12^ The distances from furcation to lingual undercut in the present study were significantly lower at second molar sites (11.04 mm vs. 14.01 mm), giving them a higher risk of lingual plate perforation. For example, if a 12-mm-long IMI were to be placed into the ISB of either molar, there would be a theoretical 42.9% risk of perforation at the second molar site vs. 16.5% at the first molar site. Likewise, there is a greater risk of damaging the IAN when IMIs are placed into inter-septal bone at mandibular second molars (furcation to IAN, 12.97 mm vs. 15.07 mm). However, an even greater risk of damaging the IAN exists when IMIs of inappropriate length are placed into molar root sockets rather than into ISB, the greatest risk being at the distal root of the second molar where the mean distal root apex-to-canal distance is only 3.84 mm, much less than the 6 mm required as a margin for safety.^13^

**Figure 9:**
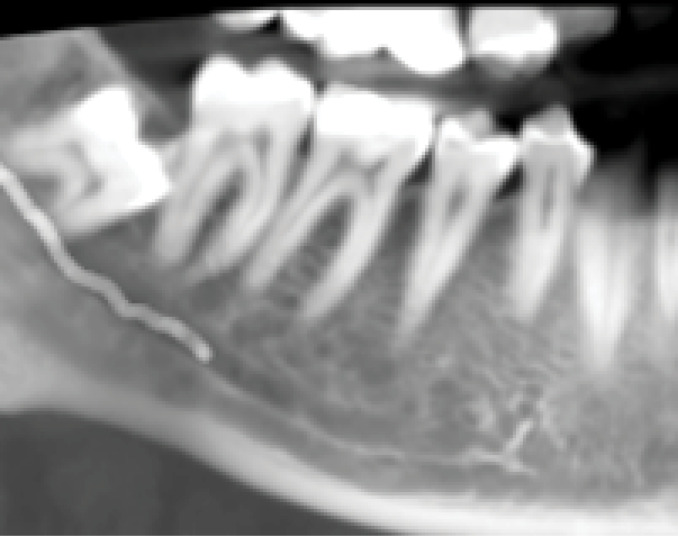
A radiograph showing a typical finding of the relative mesiodistal dimensions of ISBs at first vs. second mandibular molars

 Regarding the dimensions of ISB for both tooth sites, both the buccolingual and mesiodistal ISB widths at first molars were significantly greater than at second molars (*P* < 0.05; Figure 9), making the latter less often suitable for IMI placement into ISB and confirming that a different IMI approach may be required here.^14^ If the mesiodistal width of a molar ISB is > 2.5 mm, it could be expanded to receive an IMI using osseodensification burs in reverse mode.^5^ In our sample, we estimated that 37.2% of mandibular first molar ISBs might be suitable with this approach vs. 17.3% of second molar sites. The estimation was based on calculating the proportion of cases where the mesiodistal width of the ISB exceeded 2.5 mm out of the total number of cases in which the mesiodistal ISB was measured.

 Alternatively, as already noted, an IMI may be placed into one or other of the molar root sockets, but it is helpful to know the mean buccal and lingual cortical bone thicknesses here. Our data showed that lingual cortical thicknesses were always greater than buccal ones and that all thicknesses generally increased progressively from the first molar mesial root socket to the distal root socket of the second molar. Such thin buccal bone would make the selection of implant diameter important to leave gaps (“jumping distances”) of ≥ 2 mm for hard tissue grafting to avoid excessive bone remodeling that could ultimately leave the buccal implant surface denuded of bone. In contrast, the mean crestal buccal bone thicknesses at second molars ranged from 0.64 mm to 3.22 mm for mesial roots, making it more likely that a larger diameter and shorter implant could be considered here compared to one placed in the mesial root of a first molar. We can agree with the approach suggested by Chen et al^7^ that if the patient wishes to have both mandibular molars replaced with immediate implants, a safe approach would be to use the ISB at the first molar but to place the second implant in the mesial root socket of the second molar. The measurements of buccal thickness taken at the mid-root positions were also significantly different between the two molar locations (1.81 MB first molar vs. 4.78 MB second molar and 2.33 DB first molar vs. 5.08 DB second molar), which might suggest that ridge preservation^15^ and delayed implant placement were being contemplated, second molar sites with their thicker buccal cortical plates could be less likely to need SPG (socket preservation grafting) since thicker buccal bone does lead to less buccal bone resorption.^16^ This paper builds on our previous studies, which discussed the relevance of CBCT measurements for immediate maxillary molar implantation^17^ and guidelines for IMI placement.^18^

 Limitations of the present study include its retrospective design relying on existing records with potential selection bias. The CBCT data used was from a specific patient population, possibly limiting generalization to other populations and ethnic groups. Furthermore, measurements based on CBCT images may be subject to inter-observer and intra-observer variability, which could affect the reliability of the data. Furthermore, CBCT technology, while advanced, does have limitations in resolution and may not accurately capture subtle anatomical details critical for implant planning.

## Conclusion

 The current paper highlights the significant utility of CBCTs in evaluating risk factors in the immediate implantation of posterior mandibular teeth. The inter-radicular septum is an ideal location for IMI placement, but its suitability was found to be more common with first molars. However, the maximal implant lengths for IMIs placed in furcal bone at mandibular molar sites should not exceed 10 mm to minimize risks of violating the IAC. The likelihood of lingual plate perforations or IAC damage appears significantly higher in second molar sites, underscoring the need for careful preoperative evaluation and planning using CBCTs. When both the first and second molars need replacement, one approach could be to use the ISB for the first molar implant but a shorter (e.g., 8 mm) wider diameter implant in the mesial root socket of the second molar.

## Competing Interests

 The authors declare that they have no competing interests regarding authorship and/or publications of this paper.

## Ethical Approval

 The study was ethically approved under the code IR.GUMS.REC.1396.324.

 Ethical approval for the study was issued by IR.GUMS.REC.1396.324. Written informed consent was obtained from all the patients for using their scans in this study.
